# How to employ metabolomic analysis to research on functions of prebiotics and probiotics in poultry gut health?

**DOI:** 10.3389/fmicb.2022.1040434

**Published:** 2022-11-14

**Authors:** Mengjun Wu, Sanling Zuo, Giuseppe Maiorano, Przemysław Kosobucki, Katarzyna Stadnicka

**Affiliations:** ^1^Department of Animal Biotechnology and Genetics, Faculty of Animal Breeding and Biology, Bydgoszcz University of Science and Technology, Bydgoszcz, Poland; ^2^Department of Agricultural, Environmental and Food Sciences, University of Molise, Campobasso, Italy; ^3^Department of Food Analysis and Environmental Protection, Faculty of Chemical Technology and Engineering, Bydgoszcz University of Science and Technology, Bydgoszcz, Poland; ^4^Department of Geriatrics, Ludwik Rydygier Collegium Medicum in Bydgoszcz Nicolaus Copernicus University, Torun, Poland

**Keywords:** metabolomics, prebiotics, probiotics, gut health, poultry

## Abstract

Gut health can be considered one of the major, manageable constituents of the animal immunity and performance. The fast spread of intestinal diseases, and increase of antimicrobial resistance have been observed, therefore the intestinal health has become not only economically relevant, but also highly important subject addressing the interest of public health. It is expected, that the strategies to control infections should be based on development of natural immunity in animals and producing resilient flocks using natural solutions, whilst eliminating antibiotics and veterinary medicinal products from action. Probiotics and prebiotics have been favored, because they have potential to directly or indirectly optimize intestinal health by manipulating the metabolism of the intestinal tract, including the microbiota. Studying the metabolome of probiotics and gut environment, both *in vivo*, or using the *in vitro* models, is required to attain the scientific understanding about the functions of bioactive compounds in development of gut health and life lasting immunity. There is a practical need to identify new metabolites being the key bioactive agents regulating biochemical pathways of systems associated with gut (gut-associated axes). Technological advancement in metabolomics studies, and increasing access to the powerful analytical platforms have paved a way to implement metabolomics in exploration of the effects of prebiotics and probiotics on the intestinal health of poultry. In this article, the basic principles of metabolomics in research involving probiotics and probiotics are introduced, together with the overview of existing strategies and suggestions of their use to study metabolome in poultry.

## Introduction

### Intestinal health of poultry

It has been projected that the intensive animal production will grow continuously. By 2030, the consumption of poultry proteins is expected to increase by 15% rate in low income countries and by 25% in lower-middle income countries (OECD, FAO (2022)). Within this trend, 47% of the protein consumed from meat sources is expected to originate from poultry products. The immunity in poultry is tightly bound with optimal function of the gut and other systems within the organism that are biochemically connected with the intestine and it’s microbiome. Most of the biological systems have a defined, conceptual bidirectional networks referred to as gut axes (gut-brain axis, microbiota- immune axis, neuro- immune axis, etc.). The spread of intestinal diseases and many other pathological conditions in animals, have their beginning in dysbiosis.

In recent years, the demand for poultry products as a high-quality and affordable protein source for most people has increased year by year. According to the latest data on Meat consumption, the consumption of Poultry meat is 33.0 Kilograms/capita (OECD Meat consumption, 2022).^1^ However, the spread of enteric diseases has taken a financial toll on the global poultry industry. According to agricultural statistics in the early twenty-first century, broiler companies invested an average of $0.197 per broiler during the breeding process, but when payments to growers were included, they paid $1.15 per broiler (Clark et al., 2002). For example, the global economic loss caused by necrotizing enteritis has increased from 2 billion US dollars to 6 billion US dollars in 2015 (Zahoor et al., 2018). Meanwhile, food-borne diseases caused by Salmonella serovars and Campylobacter spp. can lead to food safety risks of zoonotic intestinal infections and increase economic losses (Hafez and Attia, 2020). It is associated with the known infectious agents (like *Salmonella*), but also with the emerging opportunistic pathogens including the isolates of enterococci, e.g., *Escherichia coli, Enterococcus cecorum, Enterococcus faecium*. Many of those species have potential to become vectors of antimicrobial resistance and potential threats to human and environment. An emerging danger and today challenges, had been accurately foreseen, over a decade earlier, at a time of implementing the regulations that put a ban on use of antimicrobial growth promoters (Yegani and Korver, 2008). Therefore, the challenges to identify and apply efficient strategies to naturally modulate the gut health and immunity are increasingly meaningful. Public investments and social demands for those challenges are being part of the European One Health Action Plan and the Farm to Fork Strategy, along with the regulation on the maximally restricted applications of the medicinal veterinary products and medical feed, which did come into force on 28th of January 2022 (European Parliament and of the Council (2019)).

Prebiotics (natural, indigestible dietary compounds that promote growth of probiotics) and probiotics (beneficial bacteria applied to the host animal and colonizing it’s gut), play significant role in strategies to optimize the poultry intestinal health, especially in the intensive animal production. Prebiotics, probiotics, and the metabolites of their activity, including postbiotics, are applied to the animals at different developmental stages, in feed and in water, with an aim to modulate and improve the host immunity and maintain the health of intestinal tract (Jha et al., 2020).

## Role of metabolomics in studying poultry gut health

Metabolomics has become an accessible and intensively used scientific study, and reveal metabolic composition and changes by examining small metabolites in various samples (Chung et al., 2018). The metabolome is a small-molecule intermediate in the metabolic process of biological systems, which has complex biologically meaningful regulation. For example, metabolomics can play a role in dietary assessment and identification of novel biomarkers of dietary intake (O’Sullivan et al., 2011), and studies of related metabolic profiles can be found in There is a lot of hypothetical role in future dietary assessments. While the metabolome reflects events downstream of gene expression, it is thought to be closer to the actual phenotype than proteomics or genomics. Słowińska et al. (2018) first applied metabolomics to identify metabolites that differentiate white and yellow turkey seminal plasma, differentially expressed metabolites involved in molecules and cells important for sperm physiology Function. Researchers can analyze the changes of related metabolic pathways from differences in metabolic profiles, such as those related to lipid, energy, and amino acid metabolic pathways, providing a line for the host’s physiological and metabolic transitions (Afrouziyeh et al., 2022). Therefore, analysis of metabolites in body fluids (e.g., urine, serum), feces and intestinal tissues after taking probiotics can improve the understanding as to how the gut microbiota and gut metabolome change. The composition and changes of these metabolites could reflect the host’s metabolic conditions and patterns, which help discover or interpret potential biological mechanisms (Cevallos-Cevallos et al., 2009; Mozzi et al., 2013).

Genomic (Zhang et al., 2014), transcriptomic (Xue et al., 2017) and proteomic (Simon et al., 2019) data of chicken have already been reported. However, only few detailed analyses of the chicken metabolome have been provided so far, especially in the gut stimulated by prebiotics and probiotics. In this review, basic principles and strategies in metabolomics of prebiotics and probiotics are presented, including nuclear magnetic resonance (NMR) and multiple MS-based analytical platforms for metabolomics. The review mainly focuses on the application of metabolomics approaches for the analysis of prebiotics and probiotics functions in poultry gut health.

## Main strategies and analytical techniques applied in metabolomics of prebiotics and probiotics

### Non-targeted and targeted metabolomics

**Figure 1:**
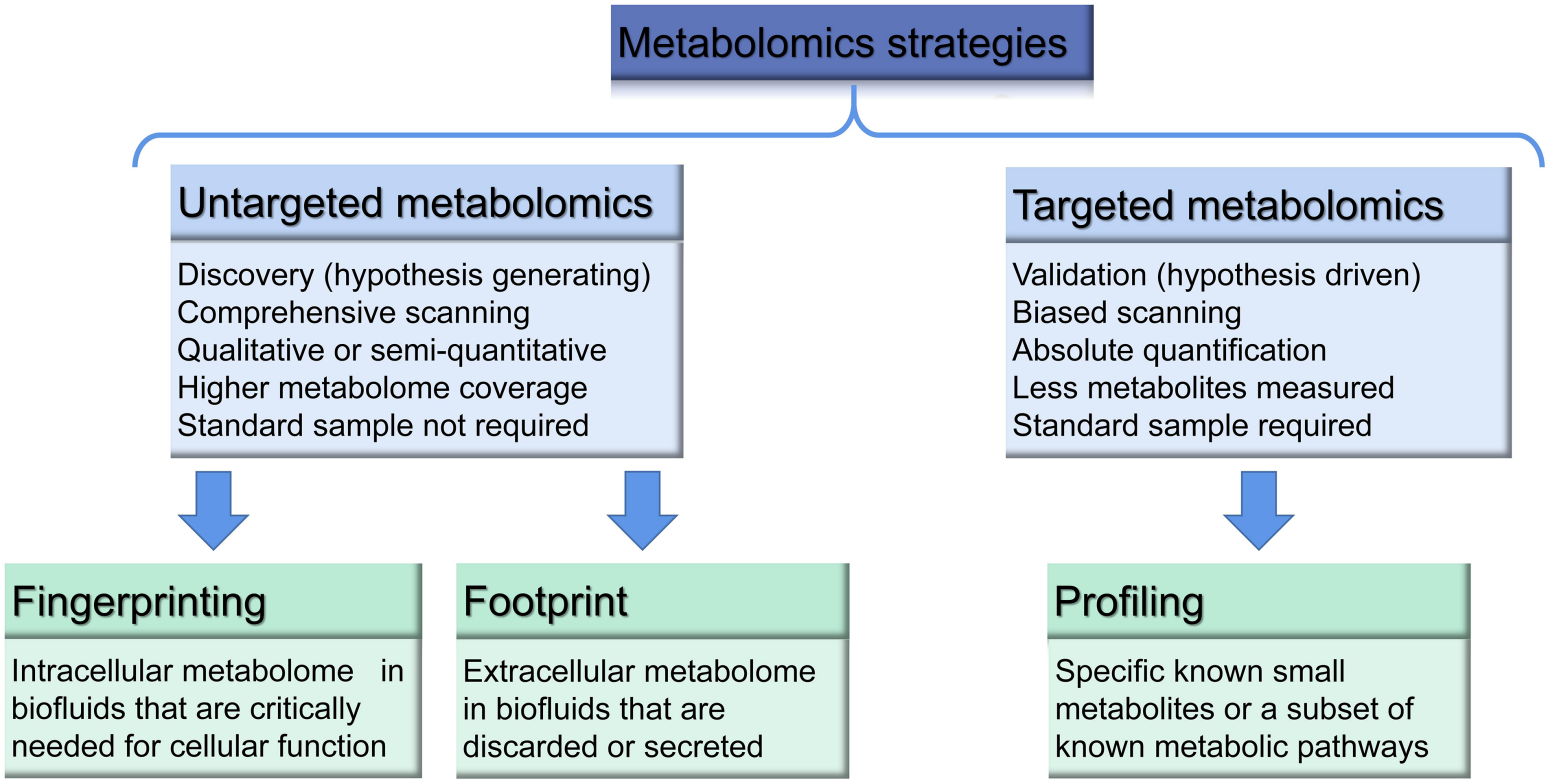
Main strategies of metabolomics modified according to literatures (Villas-Bôas et al., 2005; Klassen et al., 2017; Aszyk et al., 2018; Nalbantoglu, 2019).

The two main metabolomic strategies include hypothesis-generating and hypothesis-testing metabolomics, which are also named “non-targeted-discovery-global” and “targeted-verification-tandem” metabolomics (Nalbantoglu, 2019; Figure 1).

Non-targeted strategy is a global metabolite screening method, that allows comprehensive scanning and pattern recognition of the metabolome. It is based on the exploratory, qualitative or semi-quantitative analysis, during which the unknown metabolic identities are screened as widely as possible, with no prior knowledge of these characteristics. The main purpose of this method is to obtain an overall overview of different types of metabolites and to determine the qualitative difference between the two sets of samples (Aszyk et al., 2018). It requires development of a protocol specific for the sample, and allows to obtain a high metabolome coverage, with the number of metabolites determined. In order to systematically identify and quantify metabolites from biological samples and achieve a comprehensive characterization of biomarker targets, this analysis may cover both endometabolome (intracellular) and exometabolome (extracellular). Metabolomic fingerprinting examines the global snapshot of the intracellular metabolome to determine a general profile and classify the ingested or produced metabolites, while the metabolomic footprint analysis explores the global snapshot of the extracellular fluid metabolome (changes in cell secretions or metabolites consumed by the outer metabolome). Fingerprint and footprint analysis involve rapid analysis and usually does not require any quantification of metabolites (Villas-Bôas et al., 2005). However, due to a lack of standards, the absolute concentrations of the analytes cannot be provided, which may lead to unreliable quantification and poor repeatability.

On the contrary, a targeted metabolomics, also known as “biased or directed metabolomics” or “metabolic analysis” is referred to known standards and focused on quantitative (concentration determination) or semi-quantitative (relative intensity value evaluation) analysis of specific, acknowledged molecules/metabolites or a subset of annotated metabolic pathways (Wang et al., 2010; Klassen et al., 2017). Therefore, the targeted analysis does not necessarily require additional, extensive work for data processing but the focus on specific metabolites (Zhou and Yin, 2016). Hypothesis testing strategy of targeted metabolomics is also used to validate the results from non-targeted analysis in practice (Zhang et al., 2016). One of the types of targeted analysis is metabolome profiling, which aims to analyze a small number of metabolites in order to study biological pathways. The use of stable isotope labels ensures accurate and reliable quantification of metabolites by compensating for ion suppression effects and controlling loss of the analyte. Main drawbacks of targeted analysis are inability to identify unknown metabolites, narrow range of stable isotope labeling and the high cost (Klassen et al., 2017).

### Which metabolomic strategy to choose?

#### Non-targeted metabolomics to study function of probiotics and prebiotics

Recently, The International Scientific Association for Probiotics and Prebiotics has expanded the concept of prebiotics to include other types of compounds besides non-digestible carbohydrates, such as non-carbohydrate substances, polyphenols and certain fatty acids (e.g., polyunsaturated fatty acids), which has led to more attention put to non-targeted metabolome analysis (Bindels et al., 2015; Gibson et al., 2017; Spacova et al., 2020). There are reports on exercising non-targeted methods to obtain a comprehensive overview of altered metabolites, due to specific bioactivity of prebiotics or probiotics. E.g., production of specific bioactive metabolites was described in host organisms that utilized seaweed components as putative prebiotics (Cherry et al., 2019). In another study, the untargeted metabolomics was applied to explore probiotic survival and functionality of the bio accessible compounds in fermented camel and bovine milk after *in vitro* digestion (Ayyash et al., 2021). This method shows a discovery potential, but has also several shortcomings. Due to the large dynamic range of metabolites up to 7–9 orders of magnitude (Zhang and Powers, 2012) and sensitivity limitations, the simultaneous quantification of a large number of metabolites using MS is still challenging. If the sample contains numerous ion fragments with the same quality characteristics, unambiguous identification of bacterial metabolites may also pose a challenge. Although broad-spectrum metabolomics has a potential to reveal metabolites from the gut microbiota with an unprecedented resolution, compound quantification is extremely time-consuming, and left aside in some metabolomics programs (Klemashevich et al., 2014).

#### Feasibility of targeted metabolomics

On a contrary to drug mode of action, dietary interventions rarely have a potential to instantly block or “close” biochemical pathways or metabolic activities. Instead, they may modulate the rate of metabolite production. An accurate quantification is particularly important if subtle changes in metabolite levels are the aim of analysis. Advances in mass spectrometry (MS) instruments and methods have made the development of “targeted metabolomics” methods more accessible (Verbeke et al., 2015). With a pre-determined set of targets, it is possible to tailor extraction protocols and MS operating parameters for specific classes of metabolites to increase analytical sensitivity. E.g., phytase is one of the most common postbiotics applied in animal production, and by employing targeted metabolomics analysis, it was found as to how phytase affects specific metabolic pathways in broilers (Gonzalez-Uarquin et al., 2020). Recently, the metabolic profiling was applied in an interesting study of rapid differentiation of closely related *Lactobacilli* species. A triple quadrupole mass spectrometry (MS) was applied in combination with a linear ion trap-Orbitrap hybrid MS. The study is a good example of complementary capabilities of targeted and non-targeted metabolomics for compounds detection and their quantification in research involving closely related probiotic candidates (Yang K. et al., 2018).

**Figure 2:**
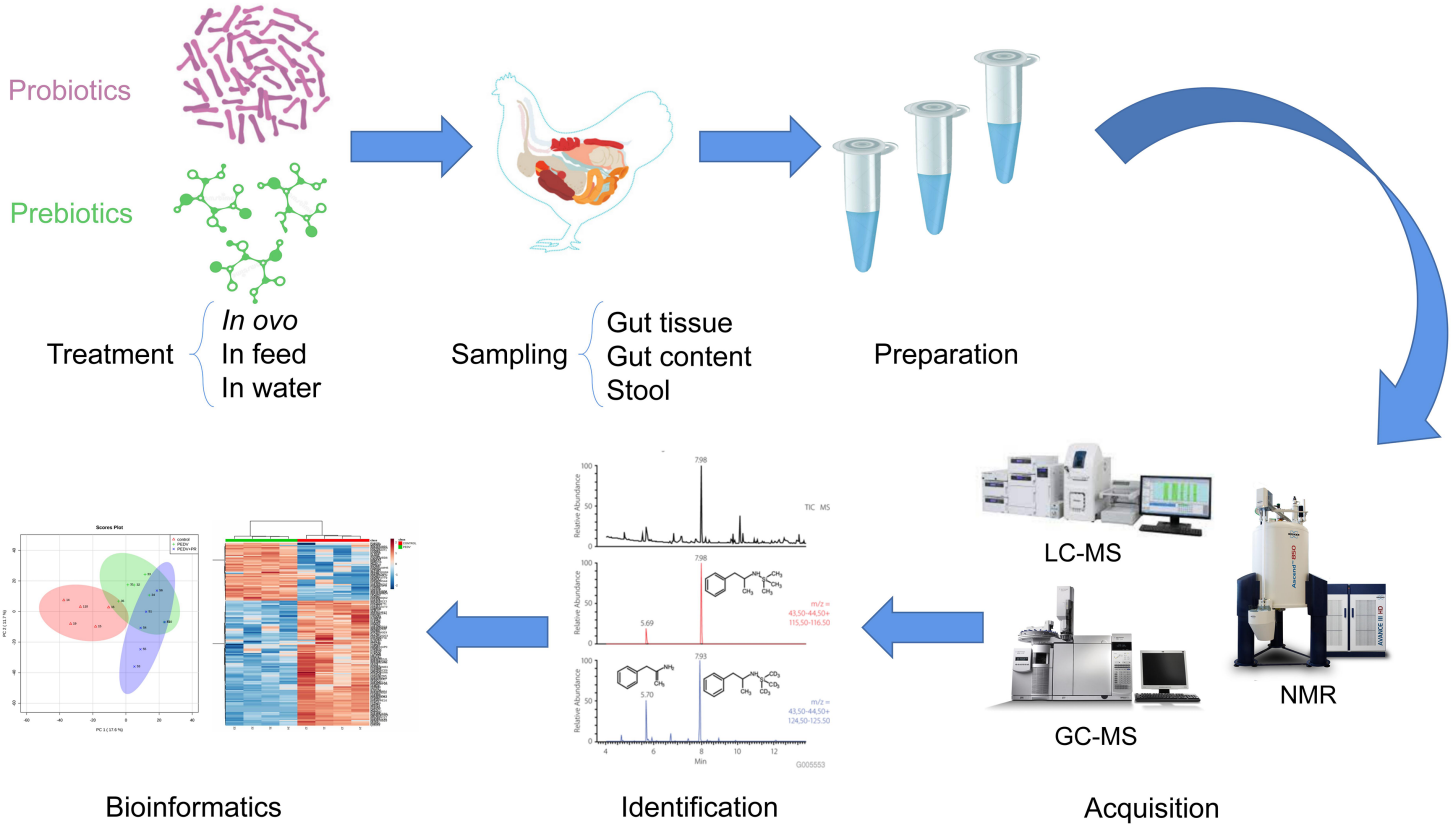
The general workflow of metabolomics analyses to study the effects of treatments with prebiotics and probiotics, in poultry.

Summarizing, adoption of a specific strategy, whether untargeted or targeted, depends on the scientific problem to be solved and the type of information that the researcher intends to obtain. The general workflow of metabolomics for prebiotics and probiotics applications in poultry is presented in Figure 2.

### Analytical platforms employed in metabolomics of prebiotics and probiotics

Various analytical instruments have been successfully adopted to metabolomics (Table 1). Nuclear magnetic resonance (NMR) is one of the most commonly used analytical platforms in metabolomics in the past decades, due to its reliability and practicality in absolute quantification (Wishart, 2009). However, NMR is relatively insensitive and the measurement range is limited to micromolar- millimolar magnitude (μM-mM). Recent advancement in development of mass spectrometry platforms including Liquid chromatography coupled to Mass spectrometry (LC–MS), Gas chromatography coupled to Mass spectrometry (GC–MS), Capillary electrophoresis–mass spectrometry (CE-MS) and Ion-mobility spectrometry–mass spectrometry (IMS-MS) provide the possibility to detect metabolites from nanomolar (nM) to picomolar (pM) concentrations, greatly improving the metabolome characterization (Goldansaz et al., 2017). Due to complexity of gut health research, the mentioned platforms can be considered complementary, providing sensitivities applicable to different molecular classes.

**Table 1:** Brief comparison of different metabolomic technologies.

Technology	Advantages	Disadvantages
NMR	Requires minimal sample preparation	Low sensitivity
	High-throughput analysis	Few numbers of metabolites
	Robust, reliable and no discriminating	Quantification challenging
	Non-destructive and direct sample analysis	
GC–MS	Mature technology	Requires sample derivatization
	Cost friendly	Long time for sample acquisition
	High reproducibility	Unable to produce parent ions
	Suitable for the detection of volatile compounds with universal databases	Difficult to identify novel compound
LC–MS	High sensitivity	Comparatively expensive
	Simple sample pretreatment	Lower reproducibility
	Wide coverage of metabolite detection	Not compatible with volatiles
	Relatively short time for sample analysis with sub-2 mm stationary phase particles	Novel compound identification is difficult

#### NMR

Nuclear magnetic resonance is a quantitative, robust and reliable technique that can be used to analyze molecular structures in biological samples, which requires minimal sample preparation, therefore relatively high-throughput analysis can be performed with this technique (Rzeznik et al., 2017). This is important for metabolomic analysis of large cohorts in animal studies. Another advantage of using NMR-based methods is that the technique is non-destructive, thus biological fluids can be preserved and allow further analysis. NMR-based metabolomics provides both structural and quantitative information, which is of great help for identifying unknown metabolites, the main bottleneck of metabolomics. NMR can simultaneously identify and quantify from dozens to hundreds of metabolites, with a detection limit of 1 μM, and has been used to characterize biological fluids in the past few decades (Emwas et al., 2019).

Despite these advantages, it must be acknowledged that NMR-based metabolomics has many limitations. Compared with mass spectrometry-based methods, the most critical limitation is the low sensitivity of this method. Despite recent advances in instrumentation, the sensitivity is still lower than that of mass spectrometry-based methods (Brennan, 2014). NMR-based metabolomics has obvious advantages in tissue metabolomics because ^1^H high-resolution magic angle rotation (HRMAS) can be used for direct sample analysis (Beckonert et al., 2010).

In monogastric animals, a study employed ^1^H NMR spectroscopy (HRMAS) to assess the effects of mouse supplementation with *Lactobacillus paracasei*, demonstrating the importance of the transgenomic, metabolic interactions between *L. paracasei* and the host to modulate the gut function, including amino-acid metabolism, methylamines and SCFAs (Martin et al., 2007, 2008). In another study, researchers used ^1^H NMR spectroscopy to characterize various tissues (including the intestine) of chicken followed by metabolite identification. In this work, around 80 metabolites were identified and utilized to develop the first chicken metabolome atlas among which only eight metabolites were found to be common for all tissue samples (Le Roy et al., 2016).

#### LC–MS

The great ability of LC to separate different compounds, from highly polar to extremely non-polar compounds, is attributed to many chromatography columns with a variety of available stationary phases (Kuehnbaum and Britz-Mckibbin, 2013). Reversed-phase chromatography and normal-phase liquid chromatography (NPLC) are traditional standard tools for the separation of non-polar, medium- polar and polar-analytes, respectively (Bieber et al., 2016; Grün and Besseau, 2016). The samples from animal like poultry contain highly polar compounds (amino acids) as well as highly hydrophobic compounds (phospholipids). Therefore, if the strategy of the research is set up for targeted metabolomics, the stationary phase can be selected according to the type of compound of interest. However, in non-targeted metabolism research, a persistent and difficult problem is that none of the current methods can comprehensively analyze all of the metabolites with different structures in a single separation. Recently, some newly developed methods like Ultra Performance Liquid Chromatography (UPLC) and hydrophilic interaction chromatography (HILIC), improved productivity and metabolome coverage (Lopes et al., 2017; Gika et al., 2019). However, if the goal is to obtain as much information as possible, more than a single type of column may be required (Rainville et al., 2014).

To meet requirements of a high resolution, rapid data acquisition and high accuracy (typically <5 ppm), the quadrupole-time-of-flight (Q-TOF) mass spectrometer, Linear trap quadrupole-Orbitrap (LTQ-Orbitrap) and Fourier transform ion cyclotron resonance (FT-ICR; Park et al., 2020) have been developed and are the most commonly employed platforms in non-targeted analysis. Instead, other mass analyzers characterized by high sensitivity and selectiveness, such as triple quadrupole (QQQ) or triple quadrupole-linear ion trap [QqQ (LIT)] mass spectrometers, are primarily dedicated to targeted analyses (Nagana Gowda and Djukovic, 2014). Due to a wide range of metabolites detectable at high resolution with R(U)PLC–MS, it has been employed for the non-targeted metabolomics of poultry intestines. Recently, the effect of dietary supplementation with *Bacillus subtilis* direct-fed microbials on chicken intestinal metabolite levels was described based on UPLC-MS global metabolomic profiling (Park et al., 2020).

#### GC–MS

Gas chromatography–mass spectrometry (GC–MS) is one of the widely used metabolomics platforms, covering both untargeted and targeted analysis. The basis of GC consists to separate volatile metabolites (or with increased volatility due to chemical derivatization), and thermally stable metabolites. GC–MS is less sensitive than LC–MS, but is generally more robust and more reproducible. Therefore, GC–MS has the potential to identify and quantify the metabolome with a higher precision and reproducibility than LC–MS (Goldansaz et al., 2017). However, unlike LC, GC typically requires chemical derivatization of the metabolic species prior to the GC–MS analysis (Nagana Gowda and Djukovic, 2014).

GC–MS is capable of analyzing less polar biomolecules involving alkyl silyl derivatives, essential oils, esters, terpenes, volatiles, carotenoids, flavonoids, and lipids, etc. Among these molecules, Volatile organic compounds (VOCs) such as fatty acids and organic acids which are important biomarker candidates in biological samples can be successfully identified by GC–MS (Nalbantoglu, 2019).

GC-TOF-MS was also commonly used to study poultry intestinal fecal metabolomics. E.g., researchers employed caecal metabolomic profiling to explore the effect of early inoculation of caecal fermentation broth, on small intestine of broilers (Gong et al., 2020). Another work adopted metabolomic analysis to study the effect of *Pediococcus acidilactici* BCC-1 and xylan oligosaccharides, in broiler chickens (Wu et al., 2021a).

### Metabolome databases and analytical pipelines with relevance to poultry species

Metabolites are identified through in-house developed, or commercial databases, such as Fiehn RTL library, MassBank, HMDB, Metlin, NIST, XCMS, Metaboanalyst, Progenesis, MetaCore, and 3Omics, etc., which are summarized in Table 2. For example, the advantage of the Fiehn library is that it contains retention index and information on retention time of the solutes, which can be compared with experiments performed according to the same analysis method (Kind et al., 2009). However, The NIST database does not contain information provided by the TOF analyzers, the high-resolution mass spectrometry; therefore, more verification steps need to be taken in data processing (Peralbo-Molina et al., 2015). Identification of metabolites also can be used in vendor software: XCalibur, MassLynx, Analyst, MassHunter, Chemstation, or Compass (Vinaixa et al., 2016).

**Table 2:** Comparison of commonly used metabolome databases.

Database	No. Records	Spectra	Metabolic pathway	Structural information	Free access	Website
NIST chemistry WebBook	31,000 compounds	MS	×	√	√	http://webbook.nist.gov/chemistry/
Golm metabolome database (GMD)	2,222 metabolites	MS	×	√	×	http://gmd.mpimp-golm.mpg.de/
Human metabolome database (HMDB)	217,920 metabolites	MS, NMR	√	√	×	http://www.hmdb.ca/
Kyoto encyclopedia of genes and genomes (KEGG)	18,920 metabolites and other small molecules	×	√	√	×	http://www.genome.jp/kegg/
Metabolite and tandem MS database (METLIN)	960,000 compounds	MS	×	√	√	http://metlin.scripps.edu/index.php
Small molecule pathway database	30,000 small molecule pathways	×	√	√	×	https://www.smpdb.ca/
Chemical entities of biological interest (ChEBI)	60,094 compounds	×	√	√	×	http://www.ebi.ac.uk/chebi/
Spectral data base (SDBS)	34,600 compounds	MS, NMR	×	√	×	http://sdbs.db.aist.go.jp/
BioCyc	20,005 pathways	×	√	√	√	http://biocyc.org
Reactome	11,291 proteins	×	√	√	×	http://www.reactome.org/
Livestock metabolome database (LMDB)	1,202 metabolites	MS	×	√	×	https://lmdb.ca/

In addition, free available software bioinformatics analysis tools available on the market can automatically perform peak selection, evaluation, and relative quantification processing, and connect the results to the metabolite database. Subsequently, data preparation workflow includes data integrity checking, data standardization, and compound name recognition (Cambiaghi et al., 2017), and further, function interpretation, enrichment analysis, pathway analysis, and metabolite pathway network diagram (KEGG, REACTOME, IPA, etc.).

### General data processing and bioinformatics analysis in metabolomics

#### Data preprocessing

**Figure 3:**
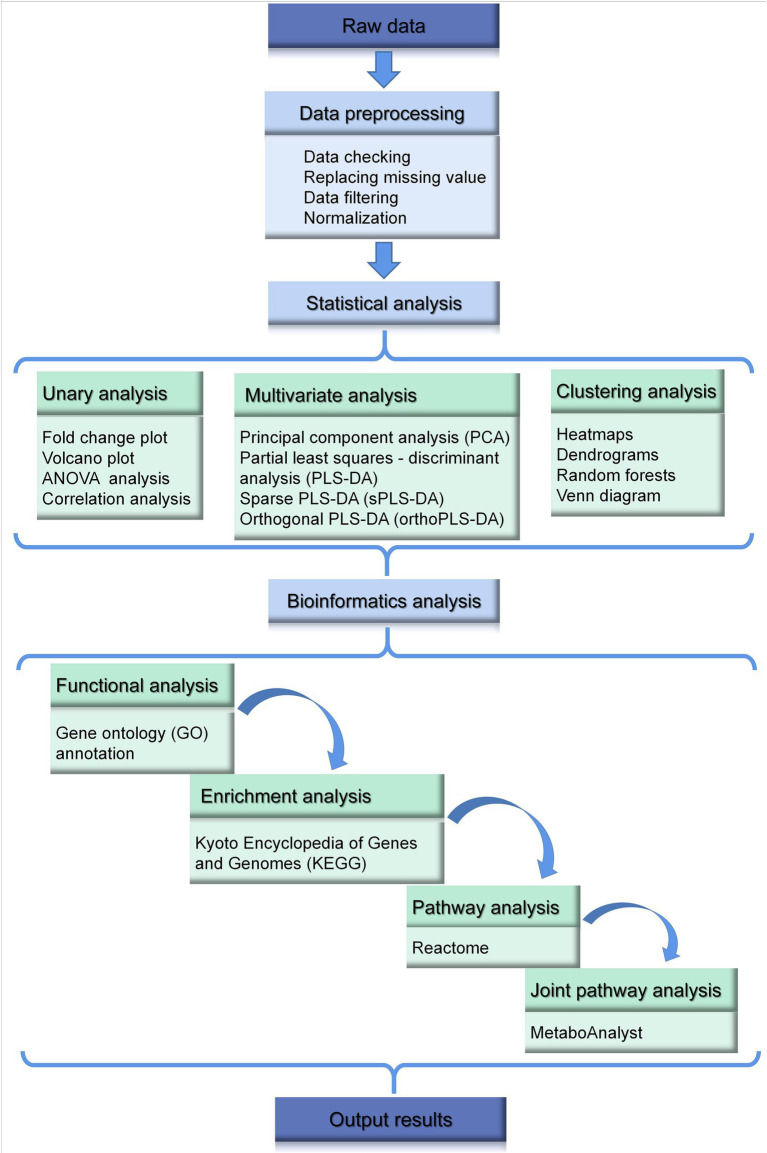
General data processing and bioinformatics analysis in metabolomics.

Common data analysis methods in metabolomics are illustrated in Figure 3, which also includes some popular and widely used bioinformatics analysis platforms. The first step of data analysis is data preprocessing. For example, on one of the most popular analytics platforms, MetaboAnalyst^2^, data integrity checking includes data checking and data filtering. Data check mainly checks whether the data format is correct, whether the classification labels are correct (at least three biological replicates are required for each group), whether it contains non-numeric data, and whether it contains missing values and indicators that are always 0. Based on this information, the basic situation of the data can be obtained, and the missing value will be replaced by a smaller value by default. Of course, MetaboAnalyst also provides more advanced programs/algorithms to deal with missing values (Xia and Wishart, 2016).

In metabolome or proteome datasets, some of the variables are caused by baseline noise and are not available in data modeling and analysis. Generally speaking, they have the following characteristics: (1) Minimal values (values near the baseline or detection limit); (2) constant values (values that do not vary with experimental conditions); (3) variables with poor reproducibility. This part of the data can be removed through data filtering functions (Chong et al., 2019).

Commonly, sample normalization can highlight the characteristics of the sample. Data conversion and data normalization mainly limit the data to a certain range, which makes subsequent analysis more convenient, and the convergence of program operation is accelerated or obeys the distribution of certain characteristic functions, so as to analyze the characteristics of the model (Pang et al., 2022). Log transformations are often employed in the metabolome and proteome (Klein, 2021).

#### Statistical analysis

Normally, statistical analysis in metabolomics of poultry usually includes unary analysis, multivariate analysis, clustering and classification analysis, variable selection, and feature selection. However, the latter two methods are less used and not discussed here.

Among unary analysis, fold change analysis and volcano plot, are typically used, to intuitively shows the difference effect (Ma et al., 2022). ANOVA and correlation analysis are also common used in analysis. Generally, differentially expressed metabolites, the possible candidate of biomarkers, are found at this step. On the other hand, more complicated multivariate analysis, regularly contains principal component analysis (PCA), partial least squares - discriminant analysis (PLS-DA), sparse partial least squares - discriminant analysis (sPLS-DA), orthogonal partial least squares - discriminant analysis (orthoPLS-DA). Unsupervised learning PCA is primarily used to discern whether there are inherent similarities and to identify possible outliers in a dataset (Saha et al., 2016). PLS-DA works well with a larger number of features than objects. For instance, an article explore changes in the metabolites of broilers supplemented with butyrate glycerides in the diet (Yang X. et al., 2018). Benefiting from the advantages of supervised learning, PLS-DA and orthoPLS-DA show a more pronounced difference than PCA. Nevertheless, even PLS are prone to fall into overfitting if the number of PLS components included in the model is larger than necessary (Liu et al., 2019).

Heatmaps and dendrograms are mostly performed during hierarchical clustering, to show the distinction of samples or/and the trend of quantities of metabolites between different samples (Wu et al., 2020). For example, a heat map was utilized to visualize the abundance of the differential metabolites in poultry treated with Galacto-oligosaccharides and xylo-oligosaccharides (Yang et al., 2022). Random forest analysis (RFA), as a supervised classification, was performed to identify metabolite signatures and the biochemical significance of the most notably altered metabolites (Park et al., 2020). The method could make biochemicals listed from bottom to top in increasing order of importance for contributing to the biochemical signatures separating the two treatment groups. In addition, venn diagrams of metabolites reveal metabolites that are commonly or uniquely regulated across groups under different conditions (Jia et al., 2020).

#### Bioinformatics analysis

Gene Ontology (GO, Gene ontology)^3^ and Kyoto Encyclopedia of Genes and Genomes Database (KEGG, Kyoto Encyclopedia of Genes and Genomes Pathways)^4^ are two classic and commonly used biomedical resource databases that provide biological function, location and pathway information of genes in multiple species. Enrichment analysis can characterize the most significantly involved metabolic terms. In addition, some other biological pathway databases such as Reactome are also an important part of the biological information database and could help to identify top-altered pathways. Recently, the research module of bioinformatics also includes joint pathway analysis, which combines data mining and biomedical research, finally could predict candidate key genes (Maity et al., 2021).

## Application of metabolomics to explain function of bioactive substances

### Metabolome characteristics under stressful conditions

In the commercial farming model, the intestinal health of poultry is very important, and many diseases can lead to the imbalance of intestinal homeostasis and thus affect the health and performance of chickens. In poultry farming, immunosuppressive diseases are caused by different diseases of the body’s immune response, affecting abnormal daily feed intake, feed conversion ratio, body weight gain, poor egg production and mortality (Li et al., 2022). *Salmonella enterica* serotype *Salmonella enteritidis* is a typical representative of non-host-specific Salmonella found in poultry, mainly through the fecal-oral route, can cause intestinal inflammation and barrier dysfunction in chickens, and has a significant impact on the poultry industry. When the feeding conditions are not good, under high temperature conditions, birds alter their behavior and physiological homeostasis to seek thermoregulation, thereby lowering their body temperature. Heat stress alters neuronal secretion profiles in birds by reducing feed intake and activating the HPA axis, thereby impairing overall poultry and egg production. Differences in the metabolome and changes in associated metabolic pathways under disease compared to normal are shown in Table 3. The latter described prebiotics and probiotics have a positive feedback on poultry metabolism.

**Table 3:** Metabolome characteristics under diseases.

Disease	Different stages	Samples	Significantly different metabolites compared to normal	Related metabolic pathways	References
Immunosuppression	Broilers	Cecal contents	2-Ketoglutaric acid		Li et al. (2022)
			Beta-glutamic acid	Cyanoamino acid metabolism	
			4-Hydroxyphenylacetic acid	Cystenie and methionine metabolism	
			Fructofuranose	Strach and sucrose metabolism	
			Gluconic acid	Glycerolipid metabolism	
			Glycyl-leucine	Aminoacyl-tRNA biosynthesis	
Salmonellosis Enteritidis	Neonatal chickens	Cecal contents	LysoPE(0:0/16:0)		Mei et al. (2021)
			3-Oxohexadecanoic acid		
			Methamphetamine	Arginine metabolism	
			Anandamide	Proline metabolism	
			Phosphocholine	Lysine biosynthesis	
			Deoxycholic acid	Lysine degradation	
			Lithocholic acid	d-Glutamate metabolism	
			L-Arabito		
Heat stress (HS)	Broilers	Plasma		Glyoxylate and dicarboxylate metabolism	Sutton (2021)
			Fumaric acid	Aspartic acid and glutamate metabolism	
			Ribitol	D-glutamine and D-glutamate metabolism	
			Succinic acid	Glycine, serine and threonine metabolism	
			Uric acid	Phenylalanine, tyrosine, and tryptophan biosynthesis	
			Mucic acid	Starch and sucrose metabolism	
			Alpha-ketoplutaric	Linoleic acid metabolism	
			2-hydroxyvaleric		
Immune stress	Broilers	Plasma	Phenyllactic acid		Bi et al. (2022a)
			3-Phenylpropanoic acid	mTOR signaling pathway	
			4-Hydroxycinnamic acid (L-phenylalanine methyl ester) amide	Apoptosis	
			Alpha-ketoglutarate	Valine, leucine, and isoleucine biosynthesis	
			N-Acetylmannosamine	Valine, leucine, and isoleucine degradation	
			Glutaric acid	Pantothenate and CoA biosynthesis	
			Alpha-ketoglutarate	Aminoacyl-tRNA biosynthesis	
Fatty liver hemorrhagic syndrome (FLHS)	Laying hens	Liver	Cytidine	Glycerophospholipid metabolism	Meng et al. (2021)
			Isomaltose	Tryptophan metabolism	
			Lysophosphatidylcholine (LysoPC) (14:0)	ARA metabolism	
			1-palmitoylglycerol	Tyrosine metabolism	
			Glutathione	Galactose metabolism	
			Lactate	Starch and sucrose metabolism	
			Glutaric acid	Biosynthesis of unsaturated fatty acids	
			Pyruvaldehyde	Phenylalanine, tyrosine and tryptophan biosynthesis	
			Tyrosine	linoleic acid metabolism	
			Uric acid	Pyruvate metabolism and glutathione metabolism	
			Arachidonic acid		
Immune stress	Broilers	Liver	5-Methylcytidine	Amino acid metabolism (valine, leucine and isoleucine biosynthesis, biosynthesis of amino acids, histidine metabolism, glycine, serine and threonine metabolism)	Bi et al. (2022b)
			(R)-3-Hydroxybutyric acid	glycerophospholipid metabolism	
			Carbofuran		
			Glycerophsphocholine		
			AICAR		
			But-2-enoic acid	Glycan metabolism (mucin type O-glycan biosynthesis, mannose type O-glycan biosynthesis)	
			Methylsuccinic acid		
			Citicoline		
			PC(18:1/14:0)	Intestinal immune network for IgA production	
			2,6-Dimethylpyrazine	Apoptosis	
			Pryruvic acid	Mannose type O-glycan biosynthesis	

### Metabolites of probiotics

**Figure 4:**
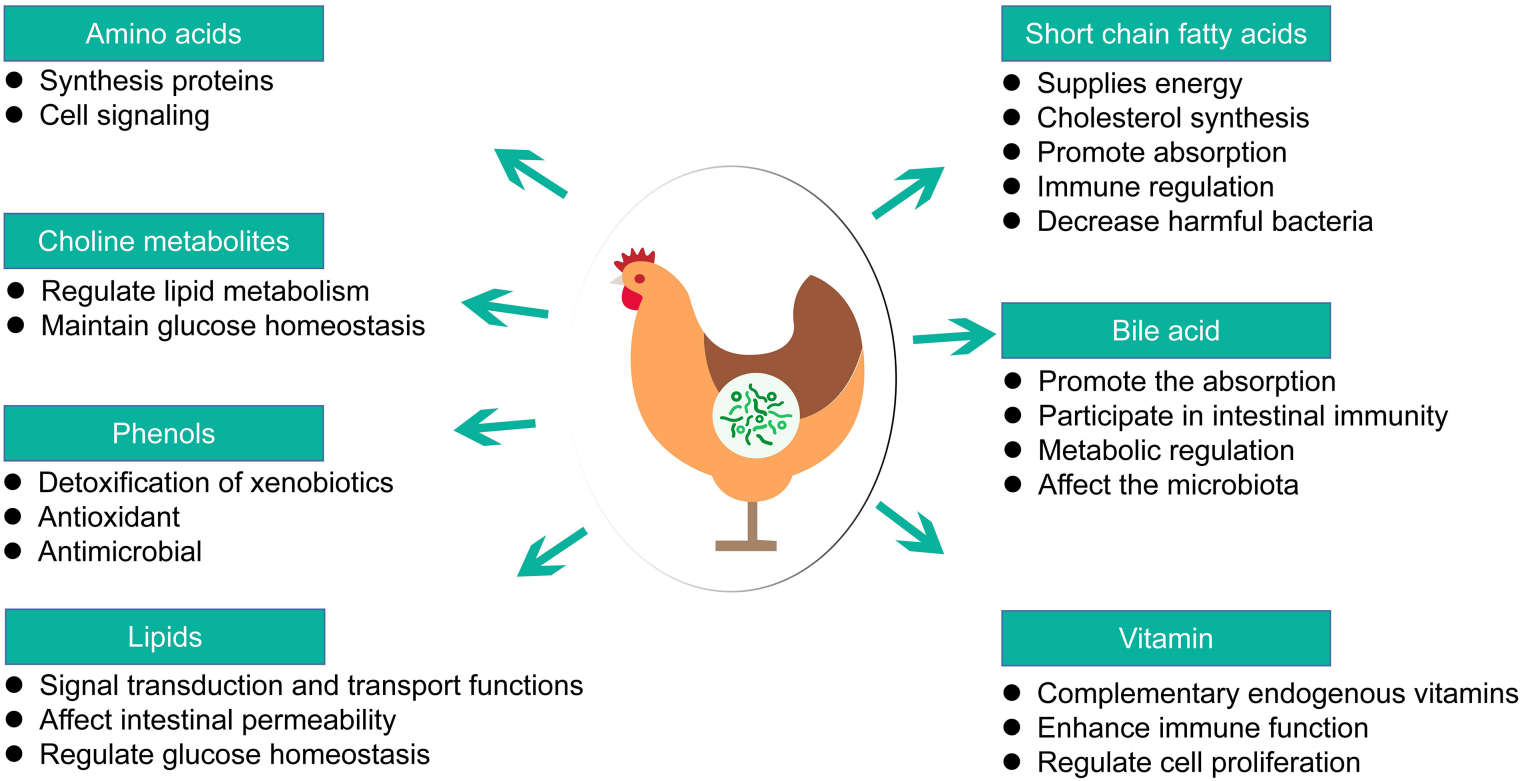
Overview of beneficial functions of metabolites produced by probiotics in poultry gut.

Probiotics are externally delivered microorganisms that colonize the intestines and exert positive health effects in the host organism, through changes in genes expression, modulating the function of immune system, and increasing resilience against environmental stressors. The beneficial functions of metabolites produced by probiotics in poultry gut were summarized in Figure 4. The examples of probiotics are: *Lactobacillus rhamnosus*, *Lactobacillus reuteri*, *Lactobacillus acidophilus*, *Bifidobacterium*, *Enterococcus faecalis* and *Clostridium butyricum*. There are numerous evidences for the key roles of metabolites produced by probiotics in contact with intestine cells, referred to as postbiotics, metabiotics (Nicholson et al., 2012). The biochemical mechanisms at the bottom of the improved gut (health) include various effects of probiotic activities leading to lower pH value in the intestines, improved absorption of calcium, iron, and vitamin D, and enhanced synthesis and absorption of multiple vitamins in the body. Some probiotics support the production of host short-chain fatty acids, cholic acids, phenols and many other metabolites, all of which are closely related to the normal or improved intestinal function, permeability and immunocompetence (Table 4). Complex carbohydrates are fermented by microorganisms in the colon into short-chain fatty acids (SCFAs), mainly acetate, propionate and butyrate, which belong to the most important products of microbial metabolism. Choline is an essential dietary nutrient, metabolized mainly in liver. Intestinal microbial enzymes can catalyze the conversion of choline into trimethylamine, which is further oxidized in the liver to produce trimethylamine N-oxide, a marker metabolite related to liver and cardiovascular diseases (Schugar and Brown, 2015). Secondary bile acids can control specific host metabolic pathways, participate in intestinal immune regulation and metabolic regulation through G protein-coupled receptors, and affect the composition of the microbial community (Vavassori et al., 2009).

**Table 4:** Metabolites produced by probiotics.

Type	Metabolites	Potential biological function	Related probiotics	References
SCFA	Acetate, propionate,butyrate, isobutyrate, 2-methylpropionate, valerate, isovalerate, hexanoate	It supplies energy for epithelial cells, participates in cholesterol synthesis, regulates the absorption of water and sodium, participates in microbe-brain-gut axis, and immune regulation.	*Bacillus subtilis; Faecalibacterium, Campylobacter jejuni*	Park et al. (2020)
				Samuel et al. (2008)
				Nothaft et al. (2017)
Lipids	Conjugated fatty acids, LPS, peptidoglycan, acylglycerols, sphingomyelin, cholesterol, phosphatidylcholines, phosphoethanolamines, triglycerides	Affect intestinal permeability, activate the brain-hepatic nerve axis in the intestine to regulate glucose homeostasis; lipopolysaccharide induces chronic systemic inflammation.	*Bifidobacterium, Lactobacillus, Enterococcus faecalis*	Nicholson et al. (2012)
				Serino et al. (2012)
				Zhang et al. (2019)
Phenolic, benzoyi and phenyl derivatives	Benzoic acid, hippuric acid, 2-hydroxyhippuric acid, 2-hydroxybenzoic acid, 3-hydroxyhippuric acid, 3-hydroxybenzoic acid, 4-hydroxybenzoic acid, 3-hydroxyphenylpropionate, 4-hydroxyphenylpropionate, 3-hydroxycinnamate, 4-methylphenol, tyrosine, phenylalanine, 4-cresol, 4-cresyl sulfate, 4-cresylglucuronide, 4-hydroxyphenylacetate, 3,4-dihydroxyphenylacetate, phenylacetylglycine, phenylacetylglutamine, phenylacetylglycine, phenylacetate, phenylpropionate, phenylpropionylglycine, cinnamoylglycine	The detoxification of xenobiotics indicates the composition and activity of intestinal microbes, using polyphenols.	*Bifidobacterium, Lactobacillus*	Zheng et al. (2011)
Bile acid	Cholate,hyocholate, deoxycholate, chenodeoxycholate, a-muricholate, b-muricholate, w-muricholate, taurocholate, glycocholate, taurochenoxycholate, glycochenodeoxycholate, taurocholate, Tauro-a-muricholate, tauro-b-muricholate, lithocholate, ursodeoxycholate, hyodeoxycholate, glycodeoxylcholate, taurohyocholate, taurodeoxylcholate	Promote the absorption of lipids and fat-soluble vitamins, participate in intestinal immunity and metabolic regulation, and affect the composition of the microbial community.	*Lactobacillus, Bifidobacteria, Enterobacter, Bacteroides, Clostridium*	Nicholson et al. (2012)
Choline metabolites	Methylamine, dimethylamine, trimethylamine, trimethylamine-N-oxide, dimethylglycine, betaine	Regulate lipid metabolism and glucose homeostasis.	*Faecalibacterium prausnitzii, Bifidobacterium*	Wang et al. (2011)
				Martin et al. (2010)
Vitamin	Vitamin K, vitamin B12, biotin, folate, thiamine, riboflavin, pyridoxine	Provide complementary sources of endogenous vitamins, enhance immune function, and exert epigenetic effects to regulate cell proliferation.	*Bifidobacterium*	Said (2011)

For example, researchers using the online software MetaboAnalyst (version 4.0)^5^ to study the effect of supplemented diets with *Bacillus subtilis* in broilers and found that it altered overall gut metabolite levels. Among these metabolites, 25 compounds significantly increased and 58 compounds significantly increased (*p* < 0.05). Pathway analysis were based on significantly different metabolites. From amino acid metabolite analysis, leucine was significantly increased, allyl alcohol (Ala-Leu), glutamyl leucine (Gln-Leu), valine (Val-Leu) and glycyl leucine (Gly-Ile) levels have roughly doubled. From the carbohydrate metabolism analysis, fructose levels increased and lactate levels decreased. Among them, glutamic acid and glutamine are mainly involved in the metabolism of carbohydrates and amino acids. As important synthetic precursors, they can promote the proliferation and maintenance of immune cells such as lymphocytes, and have an important immunomodulatory effect. Significant differences in lipid metabolism include fatty acids such as sebacate, valerylglycine, linoleoylcholine, and others. Lipid metabolites are sensed by G protein-coupled receptors (GPRs), which are present on epithelial cells and macrophages, and associated with cytokines and tight junction proteins, suggesting a role in the regulation of inflammation in the gut and the epithelium Cells are stable. The above shows that Bacillus subtilis, a probiotic, alters significantly differential metabolites in the gut, affecting amino acid, carbohydrate and fatty acid metabolism, which can be used to maintain the stability of intestinal epithelial cells and immune cells (Park et al., 2020).

### Metabolites of prebiotics’ bioactivity

Prebiotics refer to as non-digestible food components, most of which cannot be digested when passing through the digestive tract, and are used as substrates by the normal intestinal flora. Prebiotics can selectively stimulate the growth and activity of one or several specific intestinal bacteria. The most important thing is that it only stimulates the growth of beneficial bacteria, not harmful bacteria with potential pathogenicity or spoilage activity. Prebiotics increase the number of beneficial bacteria in the intestinal tract and prevent the inflammatory reaction caused by the invasion and colonization of the intestinal mucosa by *aerobic Enterobacteriaceae,* which are emerging opportunistic pathogens.

The common oligosaccharides of proven prebiotic functions, are inulin, fructooligosaccharides, galacto-oligosaccharides, isomalto-oligosaccharides and lactulose. Among them, functional oligosaccharides are the most important and the most studied type of prebiotics. Prebiotics can stimulate the growth and activity of sugar-utilizing bacteria (including *Bifidobacteriaceae* and *lactic acid bacteria*) and promote the release of organic acids. These organic acids create an antibacterial environment and inhibit the growth of intestinal pathogens. Organic acids, such as short-chain fatty acids such as acetic acid, lactic acid, propionic acid, isobutyric acid, and butyric acid, help to increase the utilization of calcium, phosphorus, and iron, promote the absorption of iron and vitamin D, acidify the intestine and reduce the abnormal fermentation caused by harmful bacteria make it difficult for the growth of pathogenic and deteriorating bacteria and consequently reduce the production of toxic compounds such as ammonia, hydrogen sulfide, indole and skatole in the metabolites of spoilage bacteria (Markowiak-Kopeć and Śliżewska, 2020).

Prebiotics selectively stimulate beneficial bacteria in the intestines and release volatile short-chain fatty acids, which lowers the pH of the intestines, making it more difficult for harmful bacteria to survive. Such changes in intestinal flora can reduce the release or expression of inflammatory transmitters, reduce disease activity index and improve intestinal mucosal damage caused by intestinal inflammation. Moreover, prebiotics can regulate the immune system of the intestine through the release or formation of organic acids, and the bacterial cell wall or cytoplasm that interacts with immune cells. The intake of oligosaccharides increases the relative abundance of beneficial bacteria such as *Bifidobacterium* and *Lactobacillus*, while the relative abundance of harmful bacteria such as *Escherichia* is reduced.

Therefore, most of the current prebiotic studies have focused on determination of the concentration of short-chain fatty acids as an important targeted metabolic test (Dunkley et al., 2007; Lei et al., 2012). The intestinal microbial metabolites involved in prebiotics which commonly used are summarized in Table 5. Fang Ma et al. found that the chicks fed with fructo-oligosaccharide (FOS) added to the feed had 93 significantly different metabolites compared with no addition, and classified them into the following 8 categories: organic heterocyclic compounds, nucleosides, nucleotides and analogs, phenylpropionic acid and polyketides, benzenes, organic oxygen compounds, organic acids and their derivatives, lipids and lipid molecules, organic nitrogen compounds (Ma et al., 2022). Differential metabolites were analyzed using one-way ANOVA and Tukey’s test. Each differential metabolite was cross-linked to a pathway in KEGG^6^, and using scipy.stats (a Python package)^7^ and use a metabolic profiler to identify the most important altered pathways and finally build. Studies have found that fructooligosaccharides(FOS) have a significant effect on the expression levels of organic matter and its derivatives, such as L-lysine, L-methionine, L-valine, L-histidine and so on. Most of these metabolites are enriched in the biosynthesis of amino groups. Amino acids are not only precursors in protein metabolism, but also intermediates in cell signaling. Protein digestion and absorption and related amino acid metabolism affect host growth. It also has the effect on the metabolism of lipids and lipid-like molecules, include 10 glycerophospholipids, 8 fatty acyl groups, 1 primary alcohol lipid, sterol and steroid derivatives. Among them, PC and PE are the most abundant phospholipids on cell membranes and play an important role in lipid metabolism and health. Glycerophospholipids are structural components of cell membranes and precursors of lipid mediators in signal transduction, suggesting that FOS is involved in the gut signal transduction and transport functions. And the arachidonic acid metabolism is an important mediator in the formation of inflammation. The down-regulated expression of coumarin and its derivatives, two isoflavones, and dihydroxybenzoates among differential metabolites indicated reduced accumulation of phenylpropionic acid and polyketides in the ileum. Phenylpropane and polyketides have multiple effects, including antioxidants, antibacterials, and anti-inflammatory agents, which may indicate reduced ileal inflammation and enhanced immune function in chickens. From the positive regulatory effect of the above FOS on the intestinal metabolism of chickens, it shows how it can improve the production, metabolism and immunity of poultry.

**Table 5:** Intestinal microbial metabolites with prebiotics.

Type	Metabolites	Potential biological function	Related prebiotics	References
SCFA	Acetate, propionate, butyrate, isobutyrate, 2-methylpropionate, valerate, isovalerate, hexanoate	Make the intestinal pH drop, more harmful bacteria difficult to survive.	Dietary fibers, Inulin, fructo-oligosaccharide, galactooligosaccharide	Dunkley et al. (2007)
				Lei et al. (2012)
				Ma et al. (2022)
Organic acids and derivatives	L-lysine, L-arginine, L-methionine, L-phenylalanine, L-histidine, L-proline, L-valine and L-citrulline	Amino acids are not only precursors of metabolic proteins, but also involved in cell signaling	Fructo-oligosaccharide	Said (2011)
Lipids	Glycerophospholipids, stearidonic acid, montecristin, cohibin C, cohibin B, DG (18:0/18:4/0:0), DG (18:3/18:3/0:0), l-hexanoylcarnitine, arachidyl carnitine, prenol lipid	PC and PE are the most abundant phospholipids in cell membrane. Glucophospholipid has a wide range of signal transduction and transport functions. Glycerophospholipids are precursors of lipid mediators in signal transduction.	Fructo-oligosaccharide	Said (2011)
Phenylpropanoids and polyketides	Gerberinol and dicoumaroylspermidine, biochanin A and daidzein, dihydroxybenzoate	Phenylpropanoids and polyketides have a variety of effects, including antioxidant (Jia et al., 2020), antimicrobial (Kępa et al., 2018) and anti-inflammatory (Doss et al.,2016).	Fructo-oligosaccharide	Said (2011)

### Metabolites of synbiotics

Synbiotics refer to the mixed products of probiotics and prebiotics, or add vitamins and trace elements. It can not only exert the physiological bacterial activity of probiotics, but also can selectively increase the number of the bacteria, so that the probiotics effect is more significant and lasting. A study analyzed the cecal metabolome of broilers, fed diet supplemented with vitamin B2, found two significant different metabolites of interest, namely short-chain fatty acids (acetic acid, propionic acid, lactic acid, lactic acid, succinic acid and butyl Acid) and metabolites related to energy metabolism (aspartic acid, glutamic acid, niacin, formic acid and pyruvate; Biagi et al., 2020).

Therefore, synbiotics has the metabolite characteristics of both probiotics and prebiotics, and is also related to energy metabolism. However, there are few relevant studies at present, and further exploration is urgently needed.

## Metabolomic study in chicken at different developmental stages

### In ovo stimulation of microbiome and gut development

Prenatal nutrition is essential for embryonic development and newborn growth, and one of the major epigenetic determinants of lifelong health. Delivery of bioactive compounds *in ovo* is an excellent model to study the embryonic development and gut health. The compounds (e.g., probiotics and prebiotics) can be optimally injected to air chamber on day 12 of egg incubation or between 18-19^th^ days, without affecting the hatchability. The last several days prior to hatching and the first week after the hatch, are the most critical period for development of chick intestine and immunity. One investigation supplemented the eggs with chitooligosaccharide (COS) and chlorella polysaccharide (CPS) on the 12.5th day of incubation and injected them into the amniotic sac of the eggs. In the collected cecal digests, short-chain fatty acids were determined by gas chromatography (Zhang et al., 2020). The metabolic pathways of microorganisms and the changes of SCFA were explored. The SCFA in the cecum were composed of acetic acid, propionic acid, isobutyric acid, butyric acid, isovaleric acid and valeric acid. COS were found to enrich the pathways of gluconeogenesis, anaerobic energy metabolism, L-isoleucine degradation, L-histidine biosynthesis and fatty acid biosynthesis. CPS enriched biosynthesis of isoprene, affected the mevalonate and fructan biosynthesis pathways, allantoin degradation and formaldehyde assimilation.

A study used a layered chick model, *in-ovo* feeding (IOF) L-arginine (Arg), and analyzed its induced metabolite changes based on LC–MS/MS metabolomics. 81 different metabolites were selected, out of which 24 different metabolites were found after the *in ovo* stimulation: 4 metabolites involved in carbohydrate metabolism, 6 related to lipid metabolism, and 4 involved in biosynthesis of primary bile acid (Dai et al., 2020).

Some researchers supplemented one-day-old male Arbor Acres plus chicks with xylan oligosaccharides (XOS) and *Pediococcus acidilactic* BCC-1. A significant increase in the content of butyric acid in the cecal chyle was observed. Differences in 32 metabolites were found, with increased concentrations of allo-inositol and 4-hydroxyphenylpyruvate. The major enriched pathways were those involved in terpene quinone-quinone biosynthesis, including ubiquinone, propionate metabolism, citrate cycle, alanine, aspartic acid and glutamate metabolism, tyrosine metabolism, arginine and proline metabolism. Microbiota and metabolome analysis has lead to assumption, that the combined supplementation of XOS and BBC-1 may have acted synergistically to reduce pathogenic bacteria, increase butyrate bacteria and promote carbohydrate fermentation (Wu et al., 2021a).

An animal experiment was conducted to study the effects of feeding comb Leghorn hens with high-fiber and non-starch polysaccharides, then the concentration of SCFA in cecum content was determined with gas–liquid chromatography (Dunkley et al., 2007). Researchers found the increased production of acetic acid was found, while the amounts of detected propionic acid and butyric acid were relatively lower. The study showed that dietary fiber components could be fermented by cecal microorganisms to form final products, such as SCFA, ammonia, CO2 and methane.

Based on non-targeted HPLC/MS metabolomics, A study explored the metabolic changes in male Ross 308 broilers, after supplementation with lauric acid (LA), a major medium-chain fatty acid (MCFA). 24 differentially produced metabolites were identified. It was found that LA significantly changed the level of the lipid compounds by down-regulating the abundance of phosphatidylcholine (PC), and increasing lysophosphatidylcholine and lysophosphatidylethylamine. Most compounds belonged to lipid and amino acid metabolism pathways, out of which the sphingolipid metabolism is the main pathway, followed by cysteine and methionine metabolism, phenylalanine metabolism, tryptophan and β-alanine metabolism. Moreover, LA also inhibits the growth of harmful bacteria to alter the host gut microbiota. So a reduction in the gut microbiota resulted in reduced levels of acetic acid, propionic acid, butyric acid, isobutyric acid and valeric acid. LA mainly modulates lipid metabolism in broilers and alters the immune-enhancing microbiota. (Wu et al., 2021b).

In another study, Ross 708 broilers were supplemented with *Bacillus subtilis*, and the metabolomics analysis was performed in ileum content. There were 30 significantly changed metabolite indicators found, among which the amino acids, peptides, lipids, vitamins, cofactors and nucleoside metabolites had the highest concentration (Park et al., 2020). Those altered metabolites were expected to maintain intestinal homeostasis in epithelial or immune cells, which may be the reason for their impact on overall intestinal health.

At present, prebiotics and probiotics are injected to the incubated eggs, and the microbiome of chickens after probiotic supplementation has been increasingly explored (Pourabedin and Zhao, 2015). It is expected that IOF has a high applicative potential to induce large-scale and life-lasting changes in structure and composition of microbial community. The knowledge about the potential of metabolic molecules driving the change of the microbiota from the perspective of metabolome, has to be established.

### Characteristics of metabolites in the gut of other avian species

Goose is often used as an animal model to study the effect of fatty liver. Metabolites in the ileum and cecum, are important players in the formation of goose fatty liver by affecting various metabolic pathways, such as glucose and fatty acid metabolism, oxidative stress and inflammation. Those pathways involve short-chain fatty acids, branched-chain amino acids and sterols, especially glycerol 3-phosphate, sphingosine, inositol, taurine, adipate, palmitic acid and cholesterol (Zhao et al., 2020).

In quails, an experiment used the UHPLC-Q-TOF/MS untargeted method to analyze blood and stool samples after supplementation of chicory. The principal component analysis (PCA) and partial least square discriminant analysis (PLS-DA) allowed for pattern recognition and identification of characteristic metabolites. The chicory supplementation showed the effect of regulating lipolysis in fat cells. Pathway enrichment analysis showed that chicory had a strong effect on quail’s glycosylphosphatidylinositol (GPI-) anchoring biosynthesis, inositol phosphate metabolism, glycerophospholipid metabolism and steroid hormone biosynthesis (Bian et al., 2018).

By supplementing *Bacillus subtilis* to turkey by direct-fed microbial (DFM), a reduced concentration of ammonia in turkey feces was found, related with high levels of branched-chain fatty acids and microbiota fermentation activity products (Tellez et al., 2020).

In another study, commercial turkeys were fed bacitracin methylene disalicylate (BMD) to commercial turkeys. Global metabolomics showed that there are more than 700 metabolites in turkey ceca (Johnson et al., 2019). The largest categories of metabolites identified were amino acid metabolites, such as tryptophan, tyramine and valine. Tryptophan is the precursor of a large number of microorganisms and host metabolites, many of which are endogenous ligands of aromatic hydrocarbon receptors (AhR), which regulate immune response and homeostasis at the intestinal epithelial level.

The Himalayan Griffin is an important reservoir of *Clostridium perfringe*ns. One recent study analyzed the gut microbiome and metabolome of this bird scavenger by means of LC–MS metabolomics. 4,490 metabolites were detected in stool samples, and 154 metabolites were identified. Among them, several metabolic compounds with important physiological functions were identified, such as 2-methylbutyrylcarnitine, 3-(phosphoacetylamino)-L-alanine, adenine, cucurbitacin B, cholic acid and N-acetyl-L-aspartic acid. The main functional categories of the meatbolites were related to carbohydrate and amino acid metabolism, replication and repair, and membrane transport (Wang et al., 2021).

Within a protection program of the wild Chinese monal (*Lophophorus lhuysii*), Jiang et al. (2020) performed non-targeted metabolomics analysis in collected stool samples and identified 58 important metabolites. These metabolites were fatty acids, bile acid derivatives, sugars and indole derivatives. Their metabolic pathways are mainly related to galactose metabolism, starch and sucrose metabolism, fatty acid biosynthesis, bile acid biosynthesis and bile secretion. A significant correlation between the fecal microbiota and metabolites was found. Major highlights of metabolomic studies performed in birds supplemented with prebiotics and probiotics in different periods of development, are presented in Table 6. Relatively little analysis of changes in the intestinal microbiome in relation to metabolomics has been performed so far, whilst the composition of microbiota is highly correlated with the composition of the metabolome.

**Table 6:** Metabolomic study in poultry species, that were supplemented with prebiotics and probiotics at various developmental timepoints.

Species	Supplementation	Important metabolites	References
In ovo feeding (Cobb 500)	Chitooligosaccharide (COS) and chlorella polysaccharide (CPS)	Short-chain fatty acids	Zhang et al. (2020)
In ovo feeding (Jinghong layers)	L-arginine	Galactose, taurine-conjugated bile acids and lipids	Dai et al. (2020)
Ross 708	*Bacillus subtilis*	Dipeptides, nucleosides, fatty acids, and carbohydrates	Park et al. (2020)
Ross 308	*Lactobacillus reuteri CSF8*	N/A	Nothaft et al. (2017)
Single comb Leghorn hens	Alfalfa crumbles	Short-chain fatty acids	Dunkley et al. (2007)
Hubbard	N/A	Volatile Fatty Acids	Lei et al. (2012)
Taiping chickens	Fructo-oligosaccharide	Organic acids and derivatives	Ma et al. (2022)
Arbor Acres plus chicks	Xylan oligosaccharides (XOS), Pediococcus acidilactic BCC-1	Sorbitol, pyridoxine, hydroxyphenyl derivatives 4-hydroxyphenylpyruvate 1 and 3-(3-hydroxyphenyl) propionic acid	Wu et al. (2021a)
Ross 308 broilers	Lauric acid (LA)	Acetic acid, propionic acid, butyric acid, isobutyric acid, valerate acid, and isovaleric acid	Wu et al. (2021b)
Chinese monal	N/A	Galactose, starch and sucrose metabolism, fatty acid, bile acid biosynthesis and bile secretion	Jiang et al. (2020)
Landes geese	N/A	Short-chain fatty acids, branched-chain amino acids, and cortisol	Zhao et al. (2020)
Nicolas turkey poults	Bacitracin methylene disalicylate	Amino acids, carbohydrates, nucleotides, peptides, and lipids	Johnson et al. (2019)
Nicholas turkey poults	Bacitracin methylene disalicylate	Indole-3-carboxylic acid, thymine,equol, 1-myristoylglycerol and pentadecanoate	Hansen et al. (2019)
Turkey	*Bacillus*	3-methylindole, p-cresol, phenol and ammonia	Tellez et al. (2020)
Japanese rock ptarmigans	N/A	Nucleic acid, free amino acids	Kobayashi et al. (2020)
Quails	Chicory	luteolin, lactucopicrin, cyanidin, taraxasterol, and β-sitosterol	Bian et al. (2018)
Shaoxing ducks	Compound probiotics	Pyridoxal (Vitamin B6), L-Arginine, and Betaine aldehyde， 7-oxocholesterol, 3-hydroxy-L-kynurenine, and N-acetyl-d-glucosamine	Sun et al. (2022)

Using metabonomics methods to detect metabolites of microbiota community in blood, feces or intestinal contents, is a way to understand mechanisms of microbiome modulation and interaction in biological systems of the host (Zhao et al., 2017).

Over a half of the published articles regarding intestinal metabolomics in poultry, focused on the chicken, followed by turkey (Table 7). Among the analyzed studies, ≤40 animals or samples per study were used to conduct the metabolomics analysis. It is worth noting that sample size did not always reflect the total number of animals used in the study. For instance, multiple samples of the same animals, but at different ages were collected and subject to analysis in some of the studies (Hansen et al., 2019; Johnson et al., 2019).

**Table 7:** Studies involving intestinal metabolomics in poultry.

Specie	Sample size	Sample	Strategy	Instrument	Number of metabolites	References
In ovo feeding (Cobb 500)	36	Cecal digesta	Targeted	GC–MS	6	Zhang et al. (2020)
In ovo feeding (Jinghong layers)	2	N/A	Untargeted	UPLC-MS	N/A	Dai et al. (2020)
Ross 708	32	Ileal contents	Untargeted	FTICR- MS	674	Park et al. (2020)
Ross 308	40	Cecal contents	Untargeted	NMR	20	Nothaft et al. (2017)
Single comb Leghorn hens	15	Cecal contents	Targeted	GC–MS	6	Dunkley et al. (2007)
Hubbard	6	Cecal contents	Targeted	GC–MS	N/A	Lei et al. (2012)
Taiping chickens	12	Ileum sample	Untargeted	LC–MS	435	Ma et al. (2022)
Arbor Acres plus chicks	40	Cecal Chyme	Untargeted	GC–MS	498	Wu et al. (2021a)
Ross 308 broilers	32	Cecal Chyme	Untargeted	GC	6	Wu et al. (2021b)
Chinese monal	9	Fecal samples	Untargeted	UHPLC–MS	323	Jiang et al. (2020)
Landes geese	24	Jejunum, ileum and cecum content	Untargeted	GC–MS	530, 589, and 657	Zhao et al. (2020)
Nicolas turkey poults	20	Cecal contents	Untargeted	UPLC–MS	712	Johnson et al., 2019
Nicholas turkey poults	30	Cecal contents	Untargeted	MALDI LTQ-Orbitrap	2000	Hansen et al. (2019)
Japanese rock ptarmigans	8	Cecal feces	Untargeted	LC–MS	116	Kobayashi et al. (2020)
Quails	32	Stool	Untargeted	LC–MS	148	Bian et al. (2018)
Shaoxing ducks	16	Cecal feces	Untargeted	LC–MS	484	Sun et al. (2022)
Himalayan Griffons	12	Stool	Untargeted	LC-MS	154	Wang et al. (2021)

The most commonly used tissue types in livestock metabolomics include cecal contents, ileum contents, and feces. The cecum is the most critical segment of the poultry intestine, where the microorganisms can hydrolyze polysaccharides, oligosaccharides and disaccharides into monosaccharides, and then further ferment them into short-chain fatty acids (SCFA). As well as gut-derived metabolites, some other biological fluids (eg blood, bile acids, not shown in table) are also used for analysis. Changes in these metabolites in the biofluids (Possible disease biomarkers) can aid to understand how functional prebiotics and probiotics affect host homeostasis in chickens (Chen et al., 2020; Zou et al., 2022). The advantage of blood samples is more fast and easy, compare to that intestinal contents or tissue. However, in terms of chicken gut study, the number of metabolites in the blood is limited and therefore the information provided is less. Biological fluids samples could be an alternative in some situations, especially when researchers focus on a certain metabolic pathway. For example, a study was performed to evaluate the effect of bile salt hydrolase inhibitors for modulating host bile profile and physiology using a chicken model system. The metabolomic analysis found that the inhibitors led to significant alterations in both circulating and intestinal bile acid signatures (Geng et al., 2020). In short, the main effects of prebiotics/probiotics intake involve increased bacterial saccharolytic activity and SCFA generation in the distal gut, It is more recommended to directly use cecal contents to study the effects of prebiotics and probiotics on gut metabolomics in poultry. Although, we still encourage researchers to use more various kinds of samples to explore thus may provide new insights into explaining the roles of prebiotics/probiotics.

## Challenges and future perspectives in studying footprint and fingerprint of probiotics activity

The majority of poultry intestinal metabolomics publications have been employing untargeted methods while fewer published studies has employed targeted strategies. This is because most of the current articles are hypothesis-generating research, the purpose is to obtain or explore as much metabolite information as possible, rather than verifying a few special metabolite information. In addition, more scientific researchers use non-targeted strategies to discover new metabolites, which will provide material for the construction of an authoritative poultry metabolome database in the future.

According to our investigation, mass spectrometry-based platforms account for most of the poultry intestinal metabolomics research. As mentioned earlier, although NMR has high reliability and practicability, in recent years, in order to detect lower concentrations of metabolites, high-resolution mass spectrometry has provided indispensable help. The number of articles using GC–MS and LC–MS to study poultry intestinal metabolomics is almost the same, showing the respective advantages of these two platforms. In addition, other non-traditional or more special mass spectrometry-based poultry intestinal metabolomics methods are also constantly being developed. These platforms include but are not limited to matrix-assisted laser desorption/ionization mass spectrometry (MALDI)-MS (Hansen et al., 2019) and Fourier transform ion cyclotron resonance (FTICR) MS (Park et al., 2020).

Another issue limiting the poultry intestinal metabolomics is the incomplete reporting of relevant background data for the metabolites identities, which are approximately from 6 to 2000, depending on the strategy used: targeted or untargeted. If possible, a good metabolomics study should include various orthogonal analytical platforms, to expand the coverage of metabolites and cross-validate the results. In most of the cases in poultry intestinal metabolomics, single analytical platforms are used, which may be due to an overlook in the experimental design, or the limited research funding. Another gap found in the analysis of the literature is the general lack of integration of the other omics analyses (proteomics, transcriptomics, and microbiome) with the metabonomics. In a light of the growing trend to study systems biology and multi-omics research, the lack of complex data integration may be considered a gap in a gut health metabolomics research.

The knowledge on the interactions of poultry intestinal cells with probiotic bacteria and metabolites their of, requires a continuous development and filling the gaps of information. The use of *in ovo* model is a very good tool to study the metabolomics of gut health in poultry. The previously established procedure allows to verify the optimal probiotic and prebiotic compounds *in vitro*, deliver them to the embryonic environment *in ovo* and track the phenotypic and genetic effects through life span of the animal (Dunislawska et al., 2017; Maiorano et al., 2017; Sobolewska et al., 2017). Therefore, the gut microbiome development is stimulated by the precise injection of probiotics and prebiotics to the air chamber, or the amnion, prior to hatch. The specific markers of the metabolic activity of probiotics can be identified *in vitro,* as so called metabolic footprints of probiotics activity, in culture medium supplemented with prebiotics. These metabolic footprints can be further explored in metabolome of a host chicken gut content/tissue, after *in ovo* injecting the selected, simple synbiotic combinations. It is proposed, that the complex picture of function of probiotics and their metabolites *in vivo* (*in ovo* model) can be complemented with tracking the metabolic footprints and fingerprints by employing new *in vitro* chicken intestine models, e.g., the Chick8E11 cell line (Khan et al., 2021), and using validated, referential intestinal *in vitro* models like the Caco-2 cell line.

The further knowledge about the function of probiotics and prebiotics in the host organism by means of metabolomic activities, is necessary to develop safe and efficient early life strategies for the pre-matured animals.

## Author contributions

MW and SZ conceived and wrote the manuscript and produced tables and figures. GM, PK, and KS conceived, supervised, and reviewed the manuscript. All authors contributed to the article and approved the submitted version.

## Funding

This work was supported by the National Science Centre UMO-2019/35/B/NZ9/03186 (OVOBIOM).

## Conflict of interest

The authors declare that the research was conducted in the absence of any commercial or financial relationships that could be construed as a potential conflict of interest.

## Publisher’s note

All claims expressed in this article are solely those of the authors and do not necessarily represent those of their affiliated organizations, or those of the publisher, the editors and the reviewers. Any product that may be evaluated in this article, or claim that may be made by its manufacturer, is not guaranteed or endorsed by the publisher.

## Footnotes

^1^ https://data.oecd.org/agroutput/meat-consumption.htm

^2^ https://www.metaboanalyst.ca/

^3^ http://www.geneontology.org

^4^ http://www.genome.ad.jp/kegg

^5^ http://www.metaboanalyst.ca/

^6^ http://www.genome.jp/kegg/

^7^ https://docs.scipy.org/doc/scipy/
